# Live probiotic cultures and the gastrointestinal tract: symbiotic preservation of tolerance whilst attenuating pathogenicity

**DOI:** 10.3389/fcimb.2014.00143

**Published:** 2014-10-15

**Authors:** Luis Vitetta, Sean Hall, Anthony W. Linnane

**Affiliations:** ^1^Sydney Medical School, The University of SydneySydney, NSW, Australia; ^2^MedlabSydney, NSW, Australia

**Keywords:** probiotics, prebiotics, synbiotics, endosymbiotic, pharmacobiotics

## Abstract

Bacteria comprise the earliest form of independent life on this planet. Bacterial development has included co-operative symbiosis with plants (e.g., *Leguminosae* family and nitrogen fixing bacteria in soil) and animals (e.g., the gut microbiome). A fusion event of two prokaryotes evolutionarily gave rise to the eukaryote cell in which mitochondria may be envisaged as a genetically functional mosaic, a relic from one of the prokaryote cells. The discovery of bacterial inhibitors such chloramphenicol and others has been exploited to highlight mitochondria as arising from a bacterial progenitor. As such the evolution of human life has been complexly connected to bacterial activity. This is embodied, by the appearance of mitochondria in eukaryotes (*alphaproteobacteria* contribution), a significant endosymbiotic evolutionary event. During the twentieth century there was an increasing dependency on anti-microbials as mainline therapy against bacterial infections. It is only comparatively recently that the essential roles played by the gastrointestinal tract (GIT) microbiome in animal health and development has been recognized as opposed to the GIT microbiome being a toxic collection of micro-organisms. It is now well-documented that the GIT microbiome is comprised of a complex cohort of commensal and potentially pathogenic bacteria. Microbial interactions in the GIT provide the necessary cues for the development of regulated signals [in part by reactive oxygen species (ROS)] that promote immunological tolerance, metabolic regulation and stability, and other factors, which may then help control local and extra-intestinal end organ (e.g., kidneys) physiology. Pharmacobiotics, the administration of live probiotic cultures is an exciting growth area of potential therapeutics, developing together with an increased scientific understanding of GIT microbiome symbiosis in health and disease. Hence probiotic bacteria may provide a therapeutic connect with the GIT microbiome that can rescue mitochondrial dysfunction by linking a biologically plausible cellular signaling program (ROS reliant) between the human host and its microbiome cohort for a continued co-operative symbiosis that maintains homeostasis favorable to both.

## Introduction

Bacteria have always conducted and orchestrated life on earth. They do so, by way of a large body of versatile metabolic activities, carrying out numerous biochemical actions such as maintaining the basic carbon, sulfur and nitrogen cycles. As such, bacteria can currently be found to inhabit multiple domains namely, terrestrial, aquatic, plant, and animal systems. The human microbiome includes not only the GIT but also all other mucosal surfaces and the skin, each with its own specific microbiome. The evolution of a primordial prokaryote fusion that led to the development of the eukaryote cell (i.e., acquisition of mitochondria) has been hypothesized as arising from an oxygen consuming bacterial ancestor (Dolan and Margulis, 2007; Shih and Matzke, 2013) by endosymbiosis; an event that highlights a significant evolutionary step, that is obligatory for the continued symbiotic existence between bacteria and plants and animals, that is vital for life and survival on this planet.

It is reported that the GIT of man harbors the densest and perhaps the most diverse concentration of bacteria known with an average of 10^12−14^ bacterial cells (predominantly in the large bowel) per wet weight of luminal content (Booijink et al., 2010). However, recent reports suggest that the skin microbiome may be just as dense and complex (Grice and Segre, 2011). Overall, there has been estimated to be 10 times more bacterial cells living on and within humans than there are human cells (Bäckhed et al., 2005).

The general scientific consensus has been that the fetus *in utero* was essentially micro-organism free indicating that post-natal infections were primordial for mucosal tissue and metabolic functionality development and maturation. However, a recent study has cast significant doubt on this notion, reporting a unique placental microbiome niche that was composed of non-pathogenic commensal bacteria similar in composition to the human oral microbiome (Aagaard et al., 2014). Hence *in utero* contact with placental commensal microbes followed by post birth amplification of bacterial cues that ensue, provide pro-inflammatory signals that induce mucosal tissue maturation. This complex activity is believed to shape immune tissue development and metabolic up-regulation that eventually provide the new born with a scaffolding of immunological tolerance and metabolic stability (Tremaroli and Bäckhed, 2012). Hence a multifaceted symbiosis is therefore established between human, bacterial, and archaeal species.

## Mitochondria and bacteria

There is nothing more central to physiological processes and biological function than its energy supply systems, the generation of exportable energy (ATP) is largely the role of the mitochondrial oxidative phosphorylation system (glycolysis being the only other significant source of net ATP synthesis) (Alberts et al., 2002). Therefore, the major role of mitochondria is cellular aerobic energy generation, and as such mitochondria have played a key role in the evolution of complex cellular systems/animals. The mitochondrial genome strongly supports the incorporation of an ancestral pre-eukaryote (a bacterial endosymbiont) (Gray et al., 1999), positing that, mitochondria arose from an endosymbiotic event through the contribution of an alpha-proteobacterium (Burger and Lang, 2003). A key discovery that supports an alpha-proteobacterium contribution to mitochondrial evolution is extant in *Rickettsia* an alpha-proteobacteria that multiplies only in eukaryotic cells.

In their studies on the origin and evolution of mitochondria Andersson et al. (1998) in effect reported that ATP production in *Rickettsia* was the same as that found in mitochondria. Andersson et al. (1998) demonstrated that *Rickettsia prowazekii* (an obligate intracellular parasite) and mitochondria have a complete set of genes and a similar collection of proteins that are involved in numerous cellular processes associated with energy production. These include, ATP production and transport, including genes encoding components of the TCA cycle, the respiratory chain complexes, the ATP-synthase complexes and the ATP/ADP translocases. Moreover, they showed that there were also some similarities in the gene orders of some of the functional clusters. Andersson et al. (1998) concluded that phylogenetic analyses signposted that *R. prowazekii* was closely related to the mitochondrion organelle than was any other microbe studied. Additional bacterial genome based research (Abhishek et al., 2011) has demonstrated matches of mitochondrial genes to those of members of the *Anaplasmataceae*, and *Rhodospirillaceae* families, showing that significant bacterial genome chimaerism had occurred en route to the formation of mitochondria in eukaryotic cells.

The mitochondrial electron transport system also produces reactive oxygen species (ROS), which through the agency of hydrogen peroxide notably regulate the redox state within cells that subsequently regulate intracellular metabolism (Linnane et al., 2007). ROS redox signaling has also been reported for the gut microbiota, giving rise to the concept that a conserved mode of cellular communication is present in bacteria (Neish, 2013) that could be perceived to mirror that, which is articulated by mitochondria in eukaryotic cells. Recently it has been further hypothesized (Vitetta et al., 2013a) that dysbiosis (a gut barrier associated abnormality that increases permeability) of the gastrointestinal tract may contribute to disease processes and their progression. Further, that pharmaceutical drug administration (e.g., analgesic medications) has been linked to gut dysbiosis (Scarpignato and Hunt, 2010).

Early electron microcopy and subcellular organelle marker enzyme studies with rat small intestine samples following the administration of a non-steroidal anti-inflammatory (NSAID) agents (i.e., indomethacin) suggested that drugs such as NSAID's induced changes in mitochondrial energy production. Hence it was reasoned that NSAID's thereby uncoupled mitochondrial oxidative phosphorylation by inducing an injurious insult on the organelle (Somasundaram et al., 1997). Animal studies have demonstrated mitochondrial morphological changes with the administration of pharmaceutical products such as NSAIDs (Leite et al., 2004).

Furthermore, maintaining the GIT ecosystem in a balanced state is a critical requisite for the control of pathogenic bacteria and the associated toxin load produced in the small and large bowel. Recent clinical trial reports with osteoarthritis patients administered analgesic medications (Coulson et al., 2012, 2013) continue to support the notion (Bengmark, 2013) that pharmacotherapy can induce adverse metabolic conditions on the gut microbial unit. This activity in turn serving to disrupt GIT homeostasis and contributing toward a dysbiotic burden that increases the risk of infective sequelae that can disrupt eukaryotic signaling and functionality.

## Commensal bacteria and conserved cellular signaling

The GIT mucosa is one of the most metabolically active tissues and attendant basic research has significantly redefined the exchanges that ensue between gut-dwelling bacteria and their vertebrate hosts. It is now very much recognized that the microbial active cohort and its mammalian host have shared not only important but critical co-evolutionary metabolic interactions that span millennia, this then serving to imprint an important endosymbiotic evolutionary event for human survival on this planet. Experiments with gnotobiotic animals (e.g., mice living in germ-free environments) has demonstrated that while the vertebrate host provides the framework genes for proper immune system functionality and therefore antigen tolerance (Falk et al., 1998), the commensal bacterial cohort provides the necessary signals that completes the process of mucosal tissue maturation.

The intestinal mucosal surface is a complex and interactional environment, which is continuously exposed to a range of commensal microorganisms. The human-microbial GIT boundary is an ecosystem that shares in a variety of important roles in human health and disease (Dominguez-Bello et al., 2010; Pepper and Rosenfeld, 2012). Inflammatory diseases of the GIT are often reported to severely disrupt the gut epithelial tight junction barrier (Qin et al., 2012; Koboziev et al., 2013) with oxidative stress purported to drive metabolic abnormalities in the gut that contribute to chronic disease development (Zhu and Li, 2012). Probiotics can affect a range of GIT physiological functions, including control over immune responses, epithelial barrier function and cellular proliferation (Vitetta et al., 2013). Recent investigations have demonstrated that some genera of human GIT bacteria can induce a rapid increase of ROS, eliciting a physiological response through the activation of epithelial NADPH oxidase-1 (Nox1) (Bermudez-Brito et al., 2012; Neish, 2013). In addition, reports site *in vitro* experiments with epithelial cells that, when co-cultured with specific probiotic bacteria, show an increased and rapid oxidation reaction of soluble redox sinks, namely glutathione and thioredoxin (Bermudez-Brito et al., 2012; Neish, 2013) that indicate the presence of a regulated process. This effect was demonstrated as an increase in the oxido-reductase reaction of transcriptional factor activations such as nuclear factor kappa B (NFκ B), NrF2 and the antioxidant response element, reflecting a cellular response to increased ROS production that is regulated (Bermudez-Brito et al., 2012; Neish, 2013). This effect must be decisive in order to elicit a restrained anti-infective response with a minimal chance of pro-inflammatory damage to the tissue. These reactions hence, define potent regulatory effects on host physiological functions that include intracellular signaling that is conserved and shared that may further define an endosymbiosis evolutionary event.

The reported mechanisms of action of probiotics are similarly aligned acting to enhance the epithelial barrier, increased bacterial adhesion to the intestinal mucosa, with an attendant inhibition of pathogen adhesion to the competitive exclusion of pathogenic microorganisms (Lee, 2008; Lin et al., 2009; Bermudez-Brito et al., 2012; Neish, 2013). Furthermore, probiotic strains have also been reported to generate a range of anti-microbial substances and to positively affect and modulate immune system function. Lee (2008) has reported that the enteric commensal bacteria by rapidly generating ROS negotiate an acceptance by the GIT epithelia. Different strains of commensal bacteria can elicit markedly different levels of ROS from contacted cells. *Lactobacilli* are especially potent inducers of ROS generation in cultured cells and *in vivo*, though all bacteria tested have some ability to alter the intracellular oxido-reductase environment (Lin et al., 2009). Yan et al. (2007) has reported that there are soluble factors that are produced by strains of *lactobacilli* that are capable of mediating beneficial effects in *in vivo* inflammatory models. This result expands our understanding of the microbiome's activity that there are ROS-stimulating bacteria that possess effective specific membrane components and or secreted factors that activate cellular ROS production to maintain homeostasis.

It has been reported that redox signaling by microbial ROS formation is in response to microbial signals via formyl peptide receptors and the gut epithelial NADPH oxidase 1 (Nox1) (Neish, 2013). As we have previously documented (Linnane et al., 2007) ROS generated by Nox enzymes have been shown to function as essential second messengers in multiple signal transduction metabolic pathways through the rapid and transient oxidative inactivation of a distinct class of sensor proteins bearing oxidant-sensitive thiol groups. These redox sensitive proteins include tyrosine phosphatases that attend as regulators of the MAP kinase pathways, focal adhesion kinase (Linnane et al., 2007; Neish, 2013). These reports focus our understanding on the importance of second messenger functionality for the maintenance of homeostasis and brings into serious question the annulment of ROS by antioxidant supplements for the amelioration of chronic diseases. The established importance of recent investigations regarding probiotic/microbial-elicited ROS teaches that stimulated cellular proliferation and motility is strictly controlled and is a regulated signaling process for proper innate immunity and gut barrier functionality (Collier-Hyams et al., 2005; Lin et al., 2009). The observations that the vertebrate epithelia of the intestinal tract, supports a tolerable low-level inflammatory response toward the GIT microflora, can be viewed as an adaptive activity that maintains homeostasis (Neish et al., 2000). This adaptive activity is an important link to an endosymbiotic event that connects eukaryotic cell function to bacteria via a conserved mode of communication that is via the upstream elaboration of ROS. Consider the following.

Aconitase is an ancient enzyme important for the Krebs cycle and is essential for mitochondrial DNA maintenance independent of its catalytic activity. An ancestral endosymbiotic event from bacteria that possessed and transmitted genes coding for aconitase may be the link that partly rationalizes the acquisition of mitochondria (mitochondrial genes) by a pre-eukaryotic ancestor. A group of bacterial endosymbionts may have added to the evolutionary step that has led to the development of mitochondria. Baughn and Malamy (2002) have suggested that a consortium of endosymbionts from the *Cytophaga-Flavobacterium-Bacteroides* phylum (of which *Bacteroides fragilis* is a member) may have contributed the aconitase gene to the nucleus of an ancestral eukaryotic cell. These endosymbionts providing a functional duality, that is control of homeostasis for growth and protection from the deleterious effects of an oxygen rich atmosphere that is analogous to the deleterious effects of oxidative stress.

Recently, Bota et al. (2002, 2005) have reported that mitochondrial aconitase is preferentially oxidatively-modified and inactivated, and that the ATP activation of the mitochondrial Lon protease specifically acts to degrade the oxidized inactivated aconitase enzyme. The authors interpret their results as demonstrating the toxicity of ROS a concept indirectly supported by Baughn and Malamy (2002). In contrast we believe that the Bota et al. (2002, 2005) conclusion is in error (Linnane et al., 2007); rather their results demonstrate how tightly-regulated is the formation of ROS and its directed activity in regulating the metabolome of the cell. The controlled specific degradation of aconitase (among the hundreds of mitochondrial proteins) to regulate the Krebs cycle's activity is an excellent example of the regulatory role that ROS play in the modulation or control of the metabolome, and that ROS do not randomly contribute to the damage or degradation of cellular metabolic processes. Rather, that an ancient endosymbiotic event that gave rise to mitochondria also evolved regulated ROS signaling pathways that are widely distributed in diverse environments from soils to commensal and probiotic bacteria found in the human gastrointestinal tract (Neish, 2013).

## Probiotics and the preservation of GIT tolerance

The mucosal surfaces and skin of vertebrates especially humans have been reported to comprise specific, complex and diverse microbial communities with distinct localized functions (Dominguez-Bello et al., 2010). The idea that live microbial cultures could enhance health and or prevent disease began to take shape in the early part of the twentieth century, when Metchnikoff (Kaufmann, 2008) postulated that the administration of lactic acid bacteria could significantly attenuate the growth and level of pathogenic bacteria in the GIT.

Gut dysbiosis an impaired GIT mucosal barrier functionality has been associated with numerous adverse health concerns such as the accumulation of endotoxins, the translocation of bacteria-derived lipopolysaccharides, and hyperactivation of the immune system. The host-microbiota through a series of complex cooperative tasks (e.g., mucus production, immune system regulation) pursues the maintenance of homeostasis. Although the clinical evidence for the benefits of probiotics is equivocal, the clinical data indicate that probiotics provide both a prophylactic and therapeutic benefit by regulating cell-signaling pathways and cytokine production. We have previously reviewed the published human studies of probiotics and prebiotics (a nutritional supplement favoring the growth and increasing the lifespan of probiotic bacteria) and their effects on several clinical scenarios (Vitetta and Sali, 2008; Vitetta et al., 2013). The probiotic strains that have been reported as clinically useful include, *Lactobacillus rhamnosus* GG, various other *Lactobacilli* and *Bifidobacteria* strains and the yeast *Saccharomyces boulardii* (Hickson, 2011). The various species of probiotics that have been clinically investigated differ from studies with single strains (e.g., *Saccharomyces boulardii, Lactobacillus rhamnosus GG, Bacillus clausii, Bifidobacterium longum, Clostridium butyricum miyairir, Lactobacillus acidophilus, Enterococcus faecium* SF68), to studies with mixtures of two or more species of probiotics, or to a synbiotic [probiotic(s) combined with a prebiotic constituent (e.g., inulin)] (Hickson, 2011).

As an example a recent meta-analysis reported the administration of probiotics for the prevention of *Clostridium Difficile* Associated Diarrhea (CDAD) (Johnston et al., 2012). The reported efficacy was for *L. rhamnosus* GG (dose: 10^9^ − 10^10^ CFU/day); *L. acidophilus* (dose: 10^9^ − 10^10^ CFU/day); *S. boulardii* lyophilized (dose: 10^9^ CFU/day); *L. plantarum* (dose: 10^9^ CFU/day); *L. acidophilus* and *L. casei* (dose: 10^9^ CFU/day) and multi-strain probiotic (dose: 10^9^ CFU/day). The duration of follow-up varied from 2 to 12 weeks, and the risk of developing CDAD was 0–24%. Hence the meta-analysis/systematic review reported that 20 randomized trials testing the effect of probiotics in patients receiving antibiotics showed a large relative risk reduction in the incidence of CDAD of 0.34 (CI, 0.24–0.49) (Johnston et al., 2012). This has provoked further interest on the therapeutic potential of the human GIT microbiome and probiotic administration. The beneficial effects occur, it would seem when the GIT environment meets a multi-strain probiotic-enhanced GIT commensal environment throughout the digestive tract.

## Conclusion

Symbiosis between the human host and its bacterial cohort provide an interaction between two different organisms living in close physical proximity that typically is advantageous to both. The genomic pool provided by the human microbiota together with the eukaryotic human nuclear genome harbor more than nine million specific genes that control a multitude of metabolic functions (Zoetendal et al., 2008). The GIT microbiome as a biosensor for the production of numerous important metabolites remains the most studied human site. As such and very likely it will provide future novel therapeutic molecular discoveries. Hence as the scientific understanding of microbial communities progresses with the important elucidation of beneficial microbial subspecies as opposed to pathogenic microbial subspecies (Vitetta and Gobe, 2013), a novel therapeutic intervention that is microbe directed and site-specific may be possible. Therefore, mechanistically probiotic bacteria may rescue mitochondrial dysfunction by linking a biologically plausible cellular signaling program (ROS dependent) between the human host and its microbiome cohort for a continued co-operative symbiosis that maintains homeostasis favorable to both.

As a final thought, the RNA molecule is a primordial molecular entity of life. The emerging evidence suggests that there are more genes encoding regulatory RNAs than those encoding proteins in the human genome (Morris and Mattick, 2014). Therefore, in the near future the role of RNAs in eukaryote cell/mitochondria evolution will need to be seriously considered.

## Author contributions

Luis Vitetta conception and design of commentary/review. Luis Vitetta, Sean Hall, and Anthony W. Linnane, read, amended, and approved the final version of the manuscript.

### Conflict of interest statement

Luis Vitetta has received National Institute of Complementary Medicine and National Health and Medical Research Council of Australia competitive funding and Industry support for research into probiotics. The authors declare that the research was conducted in the absence of any commercial or financial relationships that could be construed as a potential conflict of interest.
